# Development and Validation of a Vitamin D Status Prediction Model in Danish Pregnant Women: A Study of the Danish National Birth Cohort

**DOI:** 10.1371/journal.pone.0053059

**Published:** 2013-01-09

**Authors:** Camilla Bjørn Jensen, Andrew L. Thorne-Lyman, Linda Vadgård Hansen, Marin Strøm, Nina Odgaard Nielsen, Arieh Cohen, Sjurdur Frodi Olsen

**Affiliations:** 1 Centre for Fetal Programming, Department of Epidemiology Research, Statens Serum Institut, Copenhagen, Denmark; 2 Department of Nutrition, Harvard School of Public Health, Boston, Massachusetts, United States of America; 3 National Institute of Public Health, University of Southern Denmark, Copenhagen, Denmark; 4 Department of Clinical Biochemistry, Statens Serum Institut, Copenhagen, Denmark; Aga Khan University, Pakistan

## Abstract

Vitamin D has been hypothesized to reduce risk of pregnancy complications such as preeclampsia, gestational diabetes mellitus, and preterm delivery. However, many of these outcomes are rare and require a large sample size to study, representing a challenge for cohorts with a limited number of preserved samples. The aims of this study were to (1) identify predictors of serum 25-hydroxy-vitamin D (25(OH)D) among pregnant women in a subsample (*N* = 1494) of the Danish National Birth Cohort (DNBC) and (2) develop and validate a score predicting 25(OH)D-status in order to explore associations between vitamin D and maternal and offspring health outcomes in the DNBC. In our study sample, 42.3% of the population had deficient levels of vitamin D (<50 nmol/L 25(OH)D) and average levels of 25(OH)D-status were 56.7(s.d. 24.6) nmol/L. A prediction model consisting of intake of vitamin D from diet and supplements, outdoor physical activity, tanning bed use, smoking, and month of blood draw explained 40.1% of the variance in 25(OH)D and mean measured 25(OH)D-level increased linearly by decile of predicted 25(OH)D-score. In total 32.2% of the women were placed in the same quintile by both measured and predicted 25(OH)D-values and 69.9% were placed in the same or adjacent quintile by both methods. Cohen's weighted kappa coefficient (Κ = 0.3) reflected fair agreement between measured 25(OH)D-levels and predicted 25(OH)D-score. These results are comparable to other settings in which vitamin D scores have shown similar associations with disease outcomes as measured 25(OH)D-levels. Our findings suggest that predicted 25(OH)D-scores may be a useful alternative to measured 25(OH)D for examining associations between vitamin D and disease outcomes in the DNBC cohort, but cannot substitute for measured 25(OH)D-levels for estimates of prevalence.

## Introduction

It has long been known that vitamin D has important functions related to calcium homeostasis and bone development [1], but there has been considerable recent interest in the non-classical functions of vitamin D. Some studies have shown associations between vitamin D deficiency and certain types of cancers, heart disease, schizophrenia, type 1 diabetes, multiple sclerosis and autoimmune diseases [1], [2]. In pregnancy, vitamin D deficiency has been shown to be associated with complications such as preeclampsia, gestational diabetes mellitus, and primary caesarian section and it has been hypothesized to also induce increased risk of multiple sclerosis, heart disease, and cancer later in life [3]–[10]. However, few supplementation trials exist, and observational studies have exhibited inconsistent results for most outcomes.

Concerns have been raised that vitamin D deficiency is widespread in countries of high latitude, including Denmark [11], [12]. Therefore, it is important to understand and identify the factors that influence vitamin D status in such settings. Earlier studies have identified a number of factors that influence serum levels of 25-hydroxyvitamin D (25(OH)D), which is the generally accepted biomarker of vitamin D status [1], [13], including exposure to UVB-radiation, skin pigmentation, intake of vitamin D from diet and supplements, and certain constitutional, lifestyle and socioeconomic factors such as age, body mass index (BMI), smoking status, and education [11], [14]–[31].

A specific purpose of identifying determinants of vitamin D status is to develop algorithms that can be used to predict the vitamin D status of individuals for whom vitamin D status is not available or prohibitively expensive to estimate in the entire population. Increasingly, researchers have used “vitamin D prediction scores” constructed from variables found to influence longer term vitamin D status to explore associations with chronic disease in non-pregnant subjects [14], [17]–[19], [21], [24]–[28]. Less work has been done to apply this concept to pregnancy or to explore the validity of vitamin D predictive scores in estimating vitamin D status in the shorter term. Using data from the Danish National Birth Cohort (DNBC) [32], [33], the aims of this study were to (1) identify determinants of vitamin D status in pregnant women (2) develop and validate a prediction model of 25(OH)D-score for the purpose of exploring associations between vitamin D and maternal and offspring health outcomes in the DNBC.

## Methods

The study was performed within the DNBC; a nationwide prospective cohort study with long term follow-up [32], [33]. The cohort consisted of 101,042 pregnancies recruited in Denmark from 1996–2002. Enrolment criteria were intention to carry to term and ability to fill in questionnaires and take part in interviews in Danish. Women were enrolled at the first antenatal visit to their general practitioners. Information on lifestyle, diet, and socioeconomic status of the pregnant women was obtained from a recruitment form, a food frequency questionnaire (FFQ), and four telephone interviews (see timing of activities in **Figure S1**). Follow-up of the cohort is still ongoing.

In a previous case-control study of postpartum depression (PPD) 25(OH)D-levels were measured in 1497 pregnant women (892 non-cases and 605 cases) from blood drawn in week 25 of gestation (manuscript in preparation). The study showed no overall association between vitamin D status and risk of PPD. These data form the basis for the present study. Data were arbitrarily divided into two groups; one predictive group to develop a prediction model and one validation group to validate the model. The prediction group was randomly assigned 33% of non-cases and the validation group was assigned the remaining 67%. The prevalence of PPD is approximately 10% in the general population [34], [35], and in order to make the validation group as reflective of the overall cohort as possible we randomly allocated 66 cases of women with PPD to the validation group to reach 10% prevalence. The remaining cases were added to the prediction group to maximize the sample size in the exploratory phase. See composition of the two groups and flow chart of the study in **Figure S2**.

The blood samples were collected at the general practitioner (GP) and sent for processing and storage by regular mail. Samples were thus transported at normal temperatures for up to 48 hours, but most arrived within 28 hours. After 9–15 years in a −80°c freezer, samples were thawed and 30 µl plasma were used to analyze 25(OH)D_3_ and 25(OH)D_2_ by using “MSMS vitamin D” kit from Perkin Elmer (Waltham MA). Briefly, 30 uL of serum samples were deproteinized in microtiter plates using 120 uL acetonilrile containing ^2^H_3_-25-OH vitamin D2 and ^2^H_3_-25-OH vitamin D3 as internal standards. The supernatant was transferred to fresh plates and dried under a gentle flow of nitrogen. Subsequently the samples were derivatized using PTAD dissolved in acetonitrile. The derivatization reaction was quenched with quench solution and the samples were subjected to LC- MSMS analysis. The LC-MSMS system consisted of a CTC PAL autosampler (CTC Analytics, Zwingen, Switzerland), a Thermo surveyor LC pump and a Thermo TSQ Ultra triple quadrupole mass spectrometer (Thermo Scientific Waltham, MA). Separation was achieved using a Thermo Gold C18 column (50×2,1 mm, 3 u). The following transitions were used: 619.3/298.1 and 607.3/298.1 for 25-OH vitamin D2 and D3 respectively, 622,3/301,1 and 610,3/298,1 for internal standards of D2 and D3 respectively, 625.3/298,1 and 613,3/298,1 for the calibration standards of D2 and D3 respectively. The total coefficient of variance was 8%.

Based on existing literature [11], [14]–[31] the following potential predictors of 25(OH)D-status were selected: age (years, continuous), pre-pregnancy BMI (kg/m^2^, continuous), energy intake (MJ/day, continuous), alcohol intake (g/day, continuous, energy-adjusted by residual method), fish intake (g/day, continuous, energy-adjusted by residual method), dietary vitamin D intake (µg/day, continuous, energy-adjusted by residual method), vitamin D from supplements (µg/day, continuous), average UVB-radiation in the two months prior to blood draw (J/m^2^, continuous), month of blood draw (categorical), physical activity (min/week, continuous), parity (0, 1, 2, 3+), civil status (single, coupled/married), country of birth (Denmark, other country), socio-occupational status (high, medium, skilled, student, unskilled, unemployed), outdoor physical activity (min per week, continuous), smoking (non-smoker, occasional smoker, <15 cigarettes per day, ≥15 cigarettes per day), tanning bed use (average number of sessions per week in pregnancy before week 25, continuous), PPD case (yes, no), travels to sunny destinations during pregnancy (yes, no) (**Table S1**).

Univariate regression analyses were performed to explore associations with vitamin D status. In order to test for non-linear associations we performed spline regression models. We checked for effect modification of outdoor physical activity, tanning bed use, and travels to sunny destinations by season (winter = October to March, summer = April to September) in multivariate models consisting of 25(OH)D-level, season and the relevant variable. Variables and interaction terms with p-values<0.10 in either linear or non-linear univariate analyses were included in multivariate regression models. To develop parsimonious vitamin D scores, only variables with p-values<0.05 were retained in the final model: a stepwise approach was used to remove variables always excluding the variable with the highest p-value first. The linear term was kept in the analysis despite a p-value>0.05 if the variable had a significant non-linear term. Observations with missing values in any of the variables included in the prediction model were excluded and so were 3 observations with 25(OH)D-status >150 nmol/L because they were outliers and it was felt that they would exert disproportionate influence on the final model. (see flowchart in **Figure S2**). We compared characteristics of women who were excluded from this analysis due to missing values with those who were included and only minor differences were observed with regard to country of birth, BMI, outdoor physical activity, total physical activity and intake of vitamin D from supplements (data not shown).

The final prediction model was used to predict 25(OH)D-scores in the validation group. Predicted 25(OH)D-scores were compared to the measured 25(OH)D-levels by Pearson correlation coefficient, cross-classification in quintiles of measured 25(OH)D-levels and predicted 25(OH)D-scores, Cohen's weighted kappa coefficient, and by visual inspection of mean measured 25(OH)D-levels per decile of predicted 25(OH)D-scores.

All statistical analyses are performed in SAS for Windows version 9.3. (SAS Institute Inc., Cary, North Carolina).

## Results

Mean 25(OH)D-status was 56.7 nmol/L (std: 24.6 nmol/L, range: 5.3–145.3 nmol/L) in the total population and the 25(OH)D-levels were approximately normally distributed. As many as 10.1% had 25(OH)D-levels <25 nmol/L which is often characterized as severe vitamin D deficiency and 42.3% had 25(OH)D-levels <50 nmol/L, the threshold often used to define vitamin D deficiency. Only 23.1% had sufficient vitamin D status defined as 25(OH)D-levels >75 nmol/L. **Table 1** presents characteristics of the overall study population and of the prediction and validation groups. About half the included variables were equally distributed in the prediction group and the validation group, but mean physical activity level, outdoor physical activity level, tanning bed use, alcohol intake, 25(OH)D_2_-level, 25(OH)D_3_-level, and total 25(OH)D-level were significantly different in the prediction and validation groups. The distribution of occupational status was also significantly different in the two groups and so was the proportion of PPD-cases as this was by definition the way the groups were constructed.

**Table 1:** Characteristics of study population overall and by groups.

	Total study population		Prediction		Validation		P-value
Characteristic	N	Mean (SD)/%	N	Mean (SD)/%	N	Mean (SD)/%	
**Age** (years)	1048	29.4 (4.3)	573	29.5 (4.2)	475	29.2 (4.3)	0.8
**BMI** (kg/m^2^)	1001	23.8 (4.5)	544	23.8 (4.5)	457	23.7 (4.4)	0.6
**Parity** (children)							0.5
0	485	47.8%	256	46.4%	229	49.6%	
1	365	36.0%	209	37.9%	156	33.8%	
2	135	13.3%	73	13.2%	62	13.4%	
3+	29	2.9%	14	2.5%	15	3.3%	
**Civil status**							0.06
Single	20	2.0%	15	2.7%	5	1.1%	
Couple/married	995	98.0%	537	97.3%	458	98.9%	
**Occupational status**							0.03
High	84	8.7%	49	9.3%	35	7.9%	
Medium	295	30.5%	150	28.5%	145	32.9%	
Skilled	142	14.7%	68	12.9%	74	16.8%	
Student	90	9.3%	44	8.4%	46	10.4%	
Unskilled	222	23.0%	128	24.3%	94	21.3%	
Unemployed	134	13.9%	87	16.5%	47	10.7%	
**Physical activity** (min/week)	1428	48.6 (106.1)	797	42.8 (99.7)	631	56.0 (113.3)	0.0006
**Outdoor physical activity** (min/week)	1428	25.8 (84.5)	797	22.8 (79.1)	631	29.7 (90.8)	0.0003
**Tanning bed use** (session/week)	990	0.1 (0.2)	538	0.1 (0.2)	452	0.1 (0.2)	0.008
**Smoking**							<0.0001
Nonsmoker	743	73.2%	372	67.4%	371	80.1%	
Occasional	128	12.6%	82	14.9%	46	9.9%	
<15 cig./day	122	12.0%	82	14.9%	40	8.6%	
>15 cig./day	22	2.2%	16	2.9%	6	1.3%	
**Fish intake** (g/day)	1048	18.2 (13.9)	573	18.2 (14.4)	475	18.3 (13.3)	0.09
**Energy intake** (MJ/day)	1048	9.9 (2.5)	573	9.9 (2.5)	475	10.0 (2.5)	0.7
**Alcohol intake** (g/day)	1048	0.1 (0.7)	573	0.1 (0.8)	475	0.1 (0.5)	<0.0001
**Dietary vitamin D intake** (µg/day)	1048	3.5 (1.9)	573	3.5 (2.0)	475	3.6 (1.9)	0.3
**Vitamin D from supplements** (µg/day)	1048	6.2 (5.3)	573	6.3 (5.4)	475	5.9 (5.2)	0.4
**UVB-radiation** (J/m^2^)	1296	1025 (1027)	728	1026 (1020)	568	1024 (1036)	0.7
**Travel sunny destination**							0.5
Yes	38	2.7%	19	2.5%	19	3.1%	
No	1357	97.3%	756	97.6%	601	96.9%	
**Season of blood sample**							0.9
Summer	717	50%	403	50%	314	50%	
Winter	718	50%	401	50%	317	50%	
**PPD case**							<0.0001
Yes	605	40.5%	539	64.4%	66	90.0%	
No	889	59.5%	298	35.6%	591	10.1%	
**Vitamin D_2_ status** (nmol/L)	1484	0.7 (2.6)	835	0.7 (2.9)	649	0.6 (2.2)	<0.0001
**Vitamin D_3_ status** (nmol/L)	1484	56.1 (24.8)	835	55.7 (25.9)	649	56.5 (23.2)	0.003
**Total 25(OH)D-level** (nmol/L)	1484	56.7 (24.6)	835	56.4 (25.8)	649	57.1 (23.0)	0.002
**Country of birth**							0.08
Denmark	1442	96.5%	800	95.6%	642	97.7%	
Other countries	52	3.5%	37	4.4%	15	2.3%	

Continuous variables are given in mean (SD) and categorical variables are given in %. P-values are calculated by T-test for continuous variables and by Pearson's chi-squared test for categorical variables.

In univariate linear regression analyses the following variables were found to be significantly associated with 25(OH)D-status and were included in the prediction model: Intake of dietary vitamin D, fish intake, average UVB-radiation in the two months prior to blood draw, BMI, socio-occupational status, month of blood draw, parity, smoking, tanning-bed use, travels to sunny destinations, vitamin D intake from supplements, and maternal country of birth. A statistically significant interaction between tanning bed use and season was also found. Using bsplines with four degrees of freedom we detected non-linear associations for physical activity, energy intake, intake of dietary vitamin D, BMI, maternal age, tanning bed use, vitamin D intake from supplements and outdoor physical activity and these variables were also included in the prediction model. Further investigation indicated a quadratic relationship and hence quadratic terms were included for these variables to account for this non-linearity.

In the multivariate model variables were excluded in the following order: Fish intake, energy intake, socio-occupational status, vitamin D intake from supplements (non-linear term), physical activity, BMI, average UVB-radiation in the two months prior to blood draw, travels to sunny destinations, maternal country of birth, maternal age, and parity.

Thus the final model included the variables smoking, month of blood draw, linear and non-linear terms for dietary vitamin D intake, linear and non-linear terms for tanning bed use, the interaction term between tanning bed use and season, vitamin D intake from supplements, linear and non-linear outdoor physical activity (**Table 2**). This model explained 40.1% (R^2^ = 0.401) of the variance in 25(OH)D-status.

**Table 2:** Regression coefficients (β-estimates) from the 25(OH)D prediction model.

Parameter	Linear β estimate^1^ (nmol/L.)	95% CL^1^ (nmol/L.)	*P*-value	Non-linear β estimate^1^ (nmol/L.)	95% CL^1^ (nmol/L.)	*P*-value
**Baseline 25(OH)D-score**	21.4	11.0;31.8	<0.0001			
**Dietary vitamin D**	5.8	2.0;9.6	0.002	−0.5	−0.9;−0.1	0.01
**Vitamin D from supplements**	1.5	1.1;1.9	<0.0001			
**Tanning bed use**	63.3	31.0;95.6	<0.0001	−46.7	−81.9;−11.4	0.008
(Tanning bed use)*season						
Summer	Reference					
Winter	21.2	3.0;−39.5	0.02			
**Outdoor physical activity**	0.1	0.0;0.1	0.008	−0.0	−0.0;0	0.02
**Smoking**						
Nonsmoker	Reference					
Occasional	1.5	−3.7;6.7	0.6			
<15 cig./day	−4.6	−10.2;0.9	0.1			
>15 cig./day	−17.2	−29.0;−5.4	0.004			
**Month of blood draw**						
January	Reference					
February	0.0	−8.9;9.0	0.99			
March	3.4	−5.4;12.3	0.45			
April	6.4	−2.6;15.5	0.16			
May	14.5	6.0;23.0	0.0008			
June	19.7	10.3;29.2	<0.0001			
July	25.4	16.2;34.5	<0.0001			
August	31.6	23.0;40.1	<0.0001			
September	16.6	7.0;26.2	0.0008			
October	13.6	4.3;22.9	0.004			
November	10.8	1.9;19.6	0.02			
December	−0.5	−9.1;8.1	0.91			

1 Difference in 25(OH)D-status.

**Figure 1:**
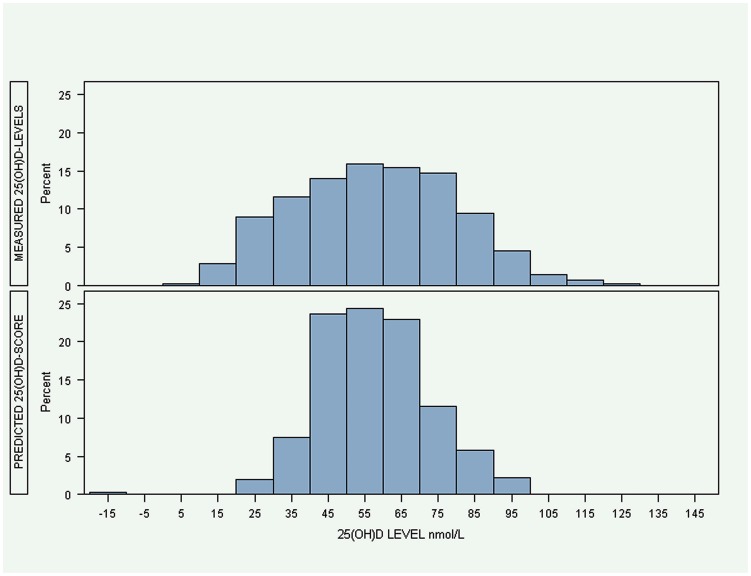
Distribution of measured 25(OH)D-levels and predicted 25(OH)D-scores.

The model was then used to predict 25(OH)D-scores in the validation group. The distribution of measured 25(OH)D-levels and predicted 25(OH)D-scores in the validation group can be seen in **Figure 1**. Most of the predicted scores fell between 20–100 nmol/L, a range that was considerably tighter than actual measured vitamin D levels. Because the model produces a 25(OH)D-score based on observed characteristics it is possible for scores to be negative despite the fact that this is biologically impossible, and this occurred for one person's estimated levels. The Pearson correlation coefficient between the measured 25(OH)D-levels and the predicted 25(OH)D-scores in the validation group was 0.4 (P<0.0001).

To determine how well the prediction model performed in ranking the individuals we cross-classified individuals from the validation group by quintiles of measured and predicted 25(OH)D-values (**Table 3**). In total 32.1% of the individuals were placed in the same quintile by both measured and predicted 25(OH)D-values, 69.9% were placed in the same or adjacent quintile by both measures, and only 1.9% were placed in opposite quintiles. Cohen's weighted kappa coefficient was 0.3 which reflects fair agreement according to Landis *et al.* 1977 [36].

**Table 3:** Cross-classification of observations in validation group by quintile of measured and predicted 25(OH)D-values.

	Quintile of measured 25(OH)D-level				
Quintile of predicted 25(OH)D-score	1	2	3	4	5
**1**	41%	30%	12%	12%	5%
**2**	30%	23%	22%	16%	10%
**3**	17%	19%	28%	14%	22%
**4**	7%	14%	20%	31%	27%
**5**	5%	13%	18%	27%	37%

**Figure 2:**
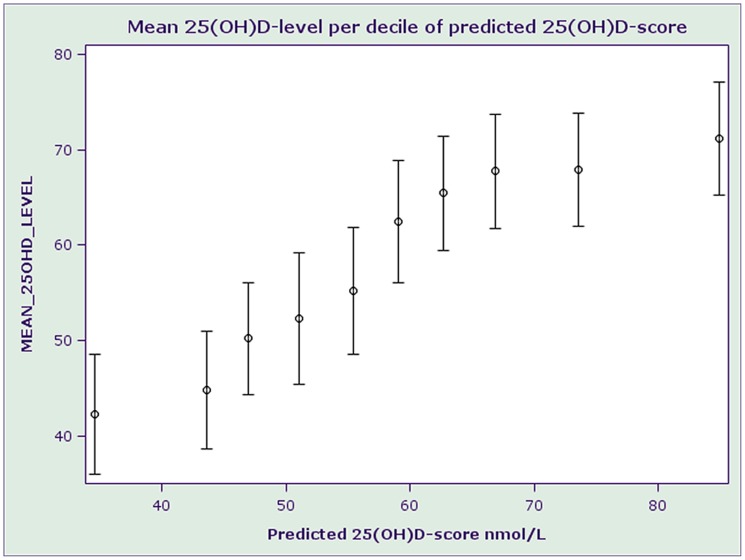
Mean 25(OH)D-level by decile of predicted 25(OH)D-score.

**Figure 2** shows that mean measured 25(OH)D-levels increased by decile of predicted 25(OH)D-score although the contrast between consecutive deciles appeared to be greater in the middle of the distribution (from 40–75 nmol/L) than at the upper or lower ends of the distribution.

## Discussion

In this subsample of the DNBC, we found high levels of vitamin D deficiency/insufficiency, evidenced by the fact that only a quarter of women had levels above 75 nmol/L. Based on a literature search we created a prediction model of 25(OH)D-score using factors previously known or suspected to influence vitamin D status. We found that 25(OH)D-scores were significantly predicted by intake of vitamin D from diet and supplements, outdoor physical activity, tanning bed use, smoking, and month of blood draw.

The final model explained 40% of the variation in 25(OH)D-status, and mean measured 25(OH)D-level increased linearly by decile of predicted 25(OH)D-score. This suggests that our vitamin D score had good ability to rank individuals according to vitamin D status. The same conclusion was reached when study participants were cross-classified according to measured and predicted 25(OH)D-status.

Other studies have presented prediction models similar to ours [14]–[31]. Similar to our findings, season of blood draw, vitamin D intake from diet and supplements, and physical activity level have previously been shown to be important predictors of 25(OH)D-score [14]–[31]. In most of these studies, both race and latitude were important predictors of vitamin D status, but these were not included in our model as we had a relatively homogenous population for both variables.

Our prediction model had a higher predictive power than most previous models reported in the literature (40.1% vs. 16–32%). [14], [15], [17]–[20], [22]–[29], [31]. These studies have generally tried to estimate longer term vitamin D status, which is a more relevant time window for the development of chronic disease outcomes such as cancer, and one might expect shorter term predictive power to be better. Our prediction model is specific to mid-pregnancy and its ability to characterize status during the first or last trimester is uncertain. While it is generally assumed that levels of 25(OH)D remain fairly constant during pregnancy, we found that season was an important determinant of vitamin D status, and might introduce misclassification error when using this score to try to predict status in other trimesters of pregnancy [37] For many pregnancy-related outcomes, and potentially later life outcomes of offspring, the window of etiological relevance of vitamin D is uncertain. making this an important issue worthy of further exploration. However, the same approach could be taken to construct scores using samples taken in early or late pregnancy.

Only one other study validated their prediction model: Bertrand *et al.* (2012) cross-classified measured and predicted 25(OH)D-status and found 59.8%–66.5% to be ranked in same or adjacent quintile and concluded that the prediction model could be used to rank individuals according to vitamin D status [14]. Also they substituted predicted score of 25(OH)D for measured 25(OH)D-status in data from a previously published study of colorectal cancer [38], and they saw similar results [14]. We have not yet performed a similar validation substitution exercise, but given that the proportion of subjects ranked in the same or adjacent quintile in our validation study was higher than in the study by Bertrand *et al.* (2012) we feel that our score could have similar utility for ranking individuals for purposes of relating predicted vitamin D status to health outcomes.

Several previous studies have shown smoking and month or season of blood draw to influence 25(OH)D-status [14]–[31]. Similar to findings of most studies, we found higher 25(OH)D-scores among nonsmokers compared with smokers. We had expected daily average UVB-radiation at and 2 months prior to the day of blood draw to be a strong predictor of 25(OH)D-score. However, in our model, month of blood measurement was a stronger predictor of status than UVB-radiation, perhaps because UVB-radiation was only collected at one site in the country, or because we lacked information on details such as use of sunscreen or extent of skin covering with clothing that may have been captured better by the seasonal variable.

While both overall physical activity and presumed outdoor physical activity were considered as potential covariates based on the findings of previous studies, only outdoor physical activity significantly predicted 25(OH)D-status. The pathway by which physical activity leads to increased skin synthesis of vitamin D is most likely through time spent outdoors and subsequent dermal synthesis of vitamin D. We looked for interactions between outdoor physical activity and season but we did not find any. Earlier studies used total physical activity as a proxy for outdoor UV exposure and one strength of our study was that we had a list of physical activities that enabled us to distinguish physical activities that were likely to have been conducted outdoors [14], [17]. However, we lacked information on other time spent outdoors, use of sunscreen, skin color and other factors known to influence dermal synthesis of vitamin D which might have improved our predictive power. Since exposure to sunlight is an important predictor of vitamin D status, and dermal synthesis of vitamin D is very limited during winter in Denmark we included travels to sunny destinations as a potential predictor of vitamin D status. To our knowledge, no other studies have included travel in their prediction analyses, and we expected to see an effect modification of season on the association between travels to sunny destinations and vitamin D status. However, only few study participants reported travelling to sunny destinations during the winter months, and data may thus have been too sparse for such associations to reach statistical significance. However, we did find that tanning bed use predicted 25(OH)D-status, and that this relationship was modified by season suggesting increased use during sun-deprived periods [23], [30].

A number of previous studies have found BMI and total body fat percentage to predict vitamin D status in non-pregnant subjects [14], [17]–[20], [22], [25], [26], [28], [29], [31]. It is thought that vitamin D, a fat soluble vitamin, may be sequestered in adipose tissue leading to reduced serum vitamin D status [1], [13]. However, pre-pregnancy BMI did not predict 25(OH)D-status in our data. This could have been due to self-reported measures of weight and height in the DNBC, and because pre-pregnancy BMI reflected another point in time compared with the blood sample.

Strengths of our study included the possibility to investigate a wide range of potential vitamin D determinants and a large sample size as well as the relatively good predictive power of the final model. As far as limitations, it is important to note that the measurement of serum vitamin D levels also involves error and is therefore an “alloyed gold standard” as far as representing actual vitamin D status of women. However, we did use liquid chromatography – tandem mass spectrometry to measure women's levels which is considered by many to be the most reliable method of assessing 25(OH)D levels. We also lacked information on ethnicity and skin color. However, our population was largely homogenous because fluency in Danish was a prerequisite for inclusion in the cohort. For this reason, and the unique availability of certain variables in our dataset, our final model is likely to be generalizable only to our specific cohort, although similar approaches could be taken to develop and validate models in other populations. We also lacked information on certain variables related to skin pigmentation, use of sunscreen or protective clothing, which might have increased the predictive power of our model. Our prediction dataset included a disproportionate number of cases of PPD because we wanted to make the best use of available data, and it is possible that this may have led to a reduction in predictive power in the validation study if this had introduced any bias. However, we found no significant association between PPD and 25(OH)D-levels in univariate analysis suggesting that this did not adversely affect the development of the score.

Blood samples were stored for 9–15 years before they were measured. Even though we used LC- MSMS analysis, which is considered the most accurate measure of vitamin D status, it is possible that there may have been some deterioration of vitamin D levels in the preserved sample. However, as noted in a recent study of 40 year old samples, if deterioration occurred, it would have led to lower levels in the entire population, and the relative differences between individuals would not be affected [39].

In conclusion, our prediction model accounted for 40.1% of the variance in vitamin D status and showed acceptable ability to properly classify individuals by quintiles of status in this cohort. 25(OH)D-status was predicted by intake of vitamin D from diet and supplements, outdoor physical activity, tanning bed use, smoking, and month of blood draw. Since prediction models only explain a proportion of the variation in 25(OH)D-status it is important to bear in mind that predicted 25(OH)D-scores cannot substitute actual blood measurements as a tool in evaluating individual vitamin D status but can be used in ranking individuals in a group according to 25(OH)D-status for purposes of examining relationships between vitamin D and disease outcomes in this cohort.

## Conclusion

In a subsample of DNBC we found 25(OH)D-scores to be predicted by intake of vitamin D from diet and supplements, outdoor physical activity, tanning bed use, smoking, and month of blood draw. Although not an ideal substitute of exact levels of vitamin D status, our prediction model showed acceptable ability of ranking individuals according to which is useful in future studies of DNBC as an alternative to vitamin D biomarkers when these are not possible to obtain due to limitied sample volumes and costs of biomarker analyses.

## Supporting Information

Figure S1 **Timing of activities in the Danish National Birth Cohort study.** Black drop indicates blood draw.
(TIF)

Figure S2 **Flow chart of the study.** ^1^Exclusion of outliers >150 nmol/L. ^2^Exclusion of observations with missing values in any variable. ^3^Exclusion of observations with missing values in any of the model variables. Cases = postpartum depression cases. Non-cases = no postpartum depression.
(TIF)

Table S1 All included variables and the source of these. CRS = The Danish Civil Registration System. DMI = Danish Meteorological Institute. GP = General Practitioner (antenatal visit week 25) DNPR = The Danish National Patient Registry.
(DOCX)

## References

[1] Holick MF (2007) Vitamin D deficiency. N Engl J Med 357: 266–281. 17634462 10.1056/NEJMra070553

[2] Holick MF, Chen TC (2008) Vitamin D deficiency: a worldwide problem with health consequences. Am J Clin Nutr 4: 1080–1086. 10.1093/ajcn/87.4.1080S 18400738

[3] Bodnar LM, Catov JM, Zmuda JM, Cooper ME, Parrott MS, et al (2010) Maternal serum 25-hydroxyvitamin D concentrations are associated with small-for-gestational age births in white women. J Nutr 140: 999–1006. 20200114 10.3945/jn.109.119636 PMC2855265

[4] Bodnar LM, Simhan HN (2010) Vitamin D may be a link to black-white disparities in adverse birth outcomes. ObstetGynecolSurv 65: 273–284. 10.1097/OGX.0b013e3181dbc55b PMC3222336 20403218

[5] Holick MF (2011) Vitamin D: A D-Lightful Solution for Health. JInvestigMed 10.231/JIM.0b013e318214ea2d PMC3738435 21415774

[6] Holmes VA, Barnes MS, Alexander HD, McFaul P, Wallace JM (2009) Vitamin D deficiency and insufficiency in pregnant women: a longitudinal study. BrJNutr 102: 876–881. 10.1017/S0007114509297236 19331703

[7] Kaludjerovic J, Vieth R (2010) Relationship between vitamin D during perinatal development and health. JMidwifery Womens Health 55: 550–560. 20974417 10.1016/j.jmwh.2010.02.016

[8] McGrath JJ, Eyles DW, Pedersen CB, Anderson C, Ko P, et al (2010) Neonatal vitamin D status and risk of schizophrenia: a population-based case-control study. ArchGenPsychiatry 67: 889–894. 10.1001/archgenpsychiatry.2010.110 20819982

[9] Thorne-Lyman A, Fawzi WW (2012) Vitamin D During Pregnancy and Maternal, Neonatal and Infant Health Outcomes: A. . Paediatr Perinat Epidemiol 26: 75–90. 22742603 10.1111/j.1365-3016.2012.01283.x PMC3843348

[10] Roth DE (2011) Vitamin D supplementation during pregnancy: safety considerations in the design. J Perinatol 31: 449–459. 21252966 10.1038/jp.2010.203

[11] Thuesen B, Husemoen L, Fenger M, Jakobsen J, Schwarz P, et al (2012) Determinants of vitamin D status in a general population of Danish adults. Bone 50: 605–610. 22227435 10.1016/j.bone.2011.12.016

[12] Husemoen LL, Thuesen BH, Fenger M, Jorgensen T, Glumer C, et al (2012) Serum 25(OH)D and Type 2 Diabetes Association in a General Population: A. . Diabetes Care Epub ahead of print 1935–5548 (Electronic).. 10.2337/dc11-1309 PMC3402265 22688545

[13] Holick MF (2011) Vitamin D: a d-lightful solution for health. J Investig Med 59: 872–880. 10.231/JIM.0b013e318214ea2d PMC3738435 21415774

[14] Bertrand KA, Giovannucci E, Liu Y, Malspeis S, Eliassen AH, et al (2012) Determinants of plasma 25-hydroxyvitamin D and development of prediction models in three US cohorts. BrJ Nutr 1–8. 10.1017/S0007114511007409 PMC3346859 22264926

[15] Davis LM, Chang SC, Mancini J, Nathanson MS, Witter FR, et al (2010) Vitamin D insufficiency is prevalent among pregnant African American adolescents. J PediatrAdolescGynecol 23: 45–52. 10.1016/j.jpag.2009.05.005 19643639

[16] Li W, Green TJ, Innis SM, Barr SI, Whiting SJ, et al (2011) Suboptimal vitamin D levels in pregnant women despite supplement use. Can J Public Health 102: 308–312. 21913590 10.1007/BF03404056 PMC6974126

[17] McCullough ML, Weinstein SJ, Freedman DM, Helzlsouer K, Flanders WD, et al (2010) Correlates of circulating 25-hydroxyvitamin D: Cohort Consortium Vitamin D Pooling Project of Rarer Cancers. AmJ Epidemiol 172: 21–35. 20562191 10.1093/aje/kwq113 PMC2892536

[18] McKinney K, Breitkopf CR, Berenson AB (2008) Association of race, body fat and season with vitamin D status among young women: a cross-sectional study. Clin Endocrinol(Oxf) 69: 535–541. 18331609 10.1111/j.1365-2265.2008.03233.x

[19] Chan J, Jaceldo-Siegl K, Fraser GE (2010) Determinants of serum 25 hydroxyvitamin D levels in a nationwide cohort of blacks and non-Hispanic whites. Cancer Causes Control 21: 501–511. 20012182 10.1007/s10552-009-9481-1 PMC3427006

[20] Hill TR, Cotter AA, Mitchell S, Boreham CA, Dubitzky W, et al (2008) Vitamin D status and its determinants in adolescents from the Northern Ireland Young Hearts 2000 cohort. BrJ Nutr 99: 1061–1067. 18197989 10.1017/S0007114507842826

[21] Hirani V, Mosdol A, Mishra G (2009) Predictors of 25-hydroxyvitamin D status among adults in two British national surveys. BrJ Nutr 101: 760–764. 18631415 10.1017/S0007114508023416 PMC3491866

[22] Rock CL, Thornquist MD, Kristal AR, Patterson RE, Cooper DA, et al (1999) Demographic, dietary and lifestyle factors differentially explain variability in serum carotenoids and fat-soluble vitamins: baseline results from the sentinel site of the Olestra Post-Marketing Surveillance Study. J Nutr 129: 855–864. 10203561 10.1093/jn/129.4.855

[23] van der Meer IM, Boeke AJ, Lips P, Grootjans-Geerts I, Wuister JD, et al (2008) Fatty fish and supplements are the greatest modifiable contributors to the serum 25-hydroxyvitamin D concentration in a multiethnic population. Clin Endocrinol(Oxf) 68: 466–472. 17941903 10.1111/j.1365-2265.2007.03066.x

[24] Gozdzik A, Barta JL, Wu H, Wagner D, Cole DE, et al (2008) Low wintertime vitamin D levels in a sample of healthy young adults of diverse. BMC Public Health 8: 1471–2458. 10.1186/1471-2458-8-336 PMC2576234 18817578

[25] Jacobs ET, Alberts DS, Foote JA, Green SB, Hollis BW, et al (2008) Vitamin D insufficiency in southern Arizona. Am J Clin Nutr 87: 608–613. 18326598 10.1093/ajcn/87.3.608 PMC4113473

[26] Lappe JM, Davies KM, Travers-Gustafson D, Heaney RP (2006) Vitamin D status in a rural postmenopausal female population. J Am Coll Nutr 25: 395–402. 17031008 10.1080/07315724.2006.10719551

[27] Egan KM, Signorello LB, Munro HM, Hargreaves MK, Hollis BW, et al (2008) Vitamin D insufficiency among African-Americans in the southeastern United. Cancer Causes Control 19: 527–535. 18219582 10.1007/s10552-008-9115-z

[28] Jacques PF, Felson DT, Tucker KL, Mahnken B, Wilson PW, et al (1997) Plasma 25-hydroxyvitamin D and its determinants in an elderly population sample. Am J Clin Nutr 66: 929–936. 9322570 10.1093/ajcn/66.4.929

[29] Andersen R, Molgaard C, Skovgaard LT, Brot C, Cashman KD, et al (2005) Teenage girls and elderly women living in northern Europe have low winter vitamin. Eur J Clin Nutr 59: 533–541. 15714215 10.1038/sj.ejcn.1602108

[30] Brot C, Vestergaard P, Kolthoff N, Gram J, Hermann AP, et al (2001) Vitamin D status and its adequacy in healthy Danish perimenopausal women: relationships to dietary intake, sun exposure and serum parathyroid hormone. BrJ Nutr 86 Suppl 1:S97–103: S97–103. 11520426 10.1079/bjn2001345

[31] Pazaitou-Panayiotou K, Papapetrou PD, Chrisoulidou A, Konstantinidou S, Doumala E, et al (2012) Height, whole Body Surface Area, gender, working outdoors, and sunbathing in previous summer are important determinants of serum 25-hydroxyvitamin D levels. Exp Clin Endocrinol Diabetes 120: 14–22. 21922453 10.1055/s-0031-1285912

[32] Olsen J, Melbye M, Olsen SF, Sorensen TI, Aaby P, et al (2001) The Danish National Birth Cohort–its background, structure and aim. Scand J Public Health 29: 300–307. 11775787 10.1177/14034948010290040201

[33] Olsen SF, Mikkelsen TB, Knudsen VK, Orozova-Bekkevold I, Halldorsson TI, et al (2007) Data collected on maternal dietary exposures in the Danish National Birth Cohort. Paediatr Perinat Epidemiol 21: 76–86. 17239183 10.1111/j.1365-3016.2007.00777.x

[34] O'hara MW, Swain AM (1996) Rates and risk of postpartum depression—a meta-analysis. Int Rev Phychiatry 8: 37–54.

[35] Kumar R (1994) Postnatal mental illness: a transcultural perspective. Soc Psychiatry PsychiatrEpidemiol 29: 250–264. 10.1007/BF00802048 7825036

[36] Landis JR, Koch GG (1977) The measurement of observer agreement for categorical data. Biometrics 33: 159–174. 843571

[37] Bruinse HW, van den Berg H (1995) Changes of some vitamin levels during and after normal pregnancy. Eur J Obstet Gynecol Reprod Biol 61: 31–37. 8549845 10.1016/0028-2243(95)02150-q

[38] Wu K, Feskanich D, Fuchs CS, Willett WC, Hollis BW, et al (2007) A nested case control study of plasma 25-hydroxyvitamin D concentrations and risk. J Natl Cancer Inst 99: 1120–1129. 17623801 10.1093/jnci/djm038

[39] Bodnar LM, Catov JM, Wisner KL, Klebanoff MA (2009) Racial and seasonal differences in 25-hydroxyvitamin D detected in maternal sera. Br J Nutr 101: 278–284. 18430263 10.1017/S0007114508981460 PMC4288959

