# Supplementary figures and images for: Development and Validation of a Vitamin D Status Prediction Model in Danish Pregnant Women: A Study of the Danish National Birth Cohort
Source: PLoS One. 2013 Jan 9;8(1):e53059. doi: 10.1371/journal.pone.0053059 (PMC3541280; doi:10.1371/journal.pone.0053059)

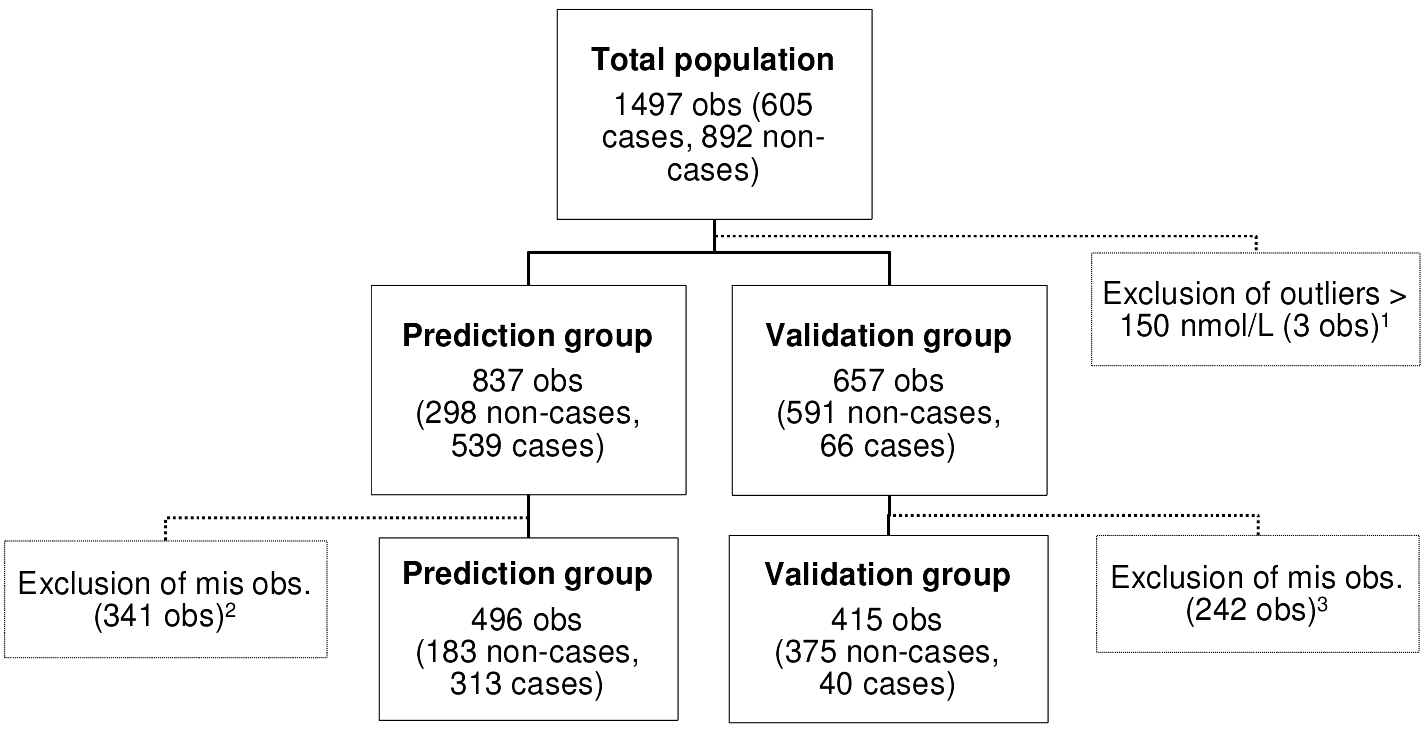

Supplement: Figure S2 — Flow chart of the study. 1Exclusion of outliers >150 nmol/L. 2Exclusion of observations with missing values in any variable. 3Exclusion of observations with missing values in any of the model variables. Cases = postpartum depression cases. Non-cases = no postpartum depression. (TIF) [file pone.0053059.s002.tif]
