# Supplementary material for: Development and Validation of a Vitamin D Status Prediction Model in Danish Pregnant Women: A Study of the Danish National Birth Cohort
Source: PLoS One. 2013 Jan 9;8(1):e53059. doi: 10.1371/journal.pone.0053059 (PMC3541280; doi:10.1371/journal.pone.0053059)
Supplement: Table S1 — All included variables and the source of these. CRS = The Danish Civil Registration System. DMI = Danish Meteorological Institute. GP = General Practitioner (antenatal visit week 25) DNPR = The Danish National Patient Registry. (DOCX) [file pone.0053059.s003.docx]

| **Source** | **FFQ**  **(week 25)** | **Interview** **1**  **(week 12)** | **Interview** **2**  **(week 30)** | **Other** |
| --- | --- | --- | --- | --- |
| Age |  |  |  | CRS |
| BMI |  | x |  |  |
| Parity |  | x |  |  |
| Civil status |  | x |  |  |
| Occupational status |  | x |  |  |
| Maternal country of birth |  |  |  | CRS |
| Physical activity level |  | x |  |  |
| Outdoor physical activity |  | x |  |  |
| Tanning bed use |  |  | x |  |
| Smoking |  | x | x |  |
| PPD case |  |  |  | DNPR |
| Fish intake | x |  |  |  |
| Energy intake | x |  |  |  |
| Alcohol intake | x |  |  |  |
| Dietary vitamin D intake | x |  |  |  |
| Vitamin D from supplements | x |  |  |  |
| Travels to sunny destinations |  | x |  |  |
| UVB-radiation |  |  |  | DMI |
| Month of blood draw |  |  |  | GP |
| Vitamin D_2_ status |  |  |  | Laboratory |
| Vitamin D_3_ status |  |  |  | Laboratory |
| Total 25(OH)D-level |  |  |  | Laboratory |
